# Supplementary material for: Exploring the Role of Glycans in the Interaction of SARS-CoV-2 RBD and Human Receptor ACE2
Source: Viruses. 2021 May 17;13(5):927. doi: 10.3390/v13050927 (PMC8156887; doi:10.3390/v13050927)
Supplement: Supplementary file 1 [file viruses-13-00927-s001.zip › viruses-1191560-supplementary.pdf]

Supporting Information:

Exploring the role of glycans in the interaction of SARS-CoV-2 RBD and human receptor ACE2

Kien Nguyen,[1,*] Srirupa Chakraborty,[1,2,*] Rachael A. Mansbach,[3,*] Bette Korber,[1] and S. Gnanakaran[1,†]

[1]*Theoretical Biology and Biophysics, Los Alamos National Laboratory, Los Alamos, NM 87545, USA*

[2]*Center for Nonlinear Studies, Los Alamos National Laboratory, Los Alamos, NM 87545, USA*

[3]*Department of Physics, Concordia University, Montreal, Quebec, Canada*

---

* These authors contributed equally to this work.

† Author to whom correspondence should be addressed. Email: gnana@lanl.gov

SUPPORTING METHODS

Choice of glycosylation and glycan modeling

The ACE2 receptor has 7 potential N-glycosylation sites (PNGS), N53, N90, N103, N322, N432, N546, and N690. Of these, the first six glycans are present in the ACE2 residue range used in our simulations (i.e. S19–D615). It remains to be seen exactly how different glycan types at these sites affect ACE2 association with the viral RBD. It is known that post-translational glycan modifications are strongly dependent on expression cell lines and their glycosylation enzyme repertoire [1, 2]. Unfortunately, all currently available ACE2 studies were done using recombinant proteins expressed in non-native cells. This prevents a definite determination of native glycosylation pattern on the ACE2 receptor and their role in RBD binding. Literature suggests that DC-SIGN and L-SIGN lectins act as enhancer factors that facilitate ACE2 mediated virus infection [3]. These specifically recognize high-mannose glycans [4], indicating that at least those glycans on ACE2 interacting with these lectins occur in oligomannose form. On the other hand, Zhao et al. [5] had previously applied sequential exo-glycosidase digestion to identify mainly biantennary N- linked glycans with sialylation and core fucosylation. Recently, Shajahan et al. [6] performed site specific mass spectrometry analysis of human ACE2 to indicate predominantly complex type glycosylation, with 60% biantennary, 85% fucosylated, and about half of them as sialated structures. Moreover, negatively charged sialic acids extensively found on complex glycans have been reported to play critical roles in viral Spike interaction [7]. A thorough understanding of the effects of glycosylation is thus necessary.

Since each PNGS can have a varying distribution of glycan occupancies [6], we modeled two divergent forms of N-glycans on the different ACE2 sites, namely the unprocessed 9-mannose (MAN9) oligomer and the enzymatically processed fucosylated 2-antennae type complex glycan (FA2) with commonly expected 2–3 linked [6] sialic acid tips. Since FA2 glycan type has been shown to be the major glycoform at the Spike glycosylation site 343 [8, 9], this was selected as the glycan choice for RBD for all glycan-included simulations. 40 initial configurations were modeled for the MAN9 and FA2 simulations (i.e. 20 for each simulation set; cf. main text Figure 1b,c). These initial configurations were prepared based on different RBD-ACE2 configurations taken from preliminary glycan-free trajectories. Glycan structures were built at the PNGS, with random orientations, using the ALLOSMOD package [10] of MODELLER [11]. This was succeeded by

short simulated annealing with the protein backbone restrained to relax the glycosylated systems at different conformations, with the CHARMM36m forcefield [12], following the glycoprotein modeling pipeline developed previously by our group [13, 14]. The CHARMM pdb and psf files were converted to AMBER format using the CHAMBER command available in the PARMED module of AMBERTOOLS 16 [15, 16]. Following the steps described, 20 different glycoprotein configurations were obtained for each of the MAN9 and FA2 glycosylated ACE2 systems, which were used for the 40 individual glycosylated trajectories of all-atom explicit-solvent simulations performed with the AMBER 16 software [15].

MM-PBSA calculations

The binding energy between RBD and ACE2 was approximated using the Molecular Mechanics Poisson-Boltzmann Surface Area (MM-PBSA) method [17, 18]. To apply this method, we used the MMPBSA.py script [19] within AMBERTOOLS 16. MM-PBSA estimates the binding energy (ΔG_{bind}) from the molecular mechanical energy (ΔE_{MM}), solvation free energy (ΔG_{sol}) and conformational entropy (ΔS) as:

$$\Delta G_{\text{bind}} = \Delta E_{\text{MM}} + \Delta G_{\text{sol}} - T\Delta S$$

with

$$\begin{aligned}\Delta E_{\text{MM}} &= \Delta E_{\text{int}} + \Delta E_{\text{vdw}} + \Delta E_{\text{ele}} \\ \Delta G_{\text{sol}} &= \Delta G_{\text{PB}} + \Delta G_{\text{SA}}\end{aligned}$$

T is the temperature; ΔE_{int} is the internal energy from the sum of bond, angle, and dihedral terms; ΔE_{vdw} is the van der Waals energy; ΔE_{ele} is the electrostatic energy; ΔG_{PB} is the electrostatic solvation free energy computed by the Poisson-Boltzmann (PB) method [20]; and ΔG_{SA} is nonpolar solvation free energy proportional to solvent accessible surface area and cavitation terms. ΔG_{SA} implicitly includes the solvent entropy approximation by virtue of parameterization [21, 22]. Because the PB calculations are computationally very costly, 4 sets of randomly selected 200 snapshots were used from the complete ensembles for these calculations in order to obtain robust sampling and standard errors.

MM-PBSA has been shown to perform reasonably well for protein-glycan systems in order to calculate relative affinity changes and their agreement with experimental values [23]. The conformational entropy change (ΔS) is not included in the present MM-PBSA analysis since entropy calculations are typically error-prone [24, 25] and have convergence difficulties [26, 27]. It has been shown that the inclusion of entropic terms from quasi-harmonic approximation provided no meaningful improvement in the agreement between the predicted and experimental energies, whereas other methods of conformational entropy calculations such as harmonic approximation entropies reduced the correlation [23]. Here, the quasi-harmonic conformational entropy was calculated from the eigenvectors of complete covariance matrix in GROMACS v5.1.2 [28] (Figure S2a). Since the relative changes in conformational entropy are similar between the MAN9 and FA2 simulations (Figure S2a), we did not included them in our binding energy calculations. Electrostatic potential calculation was performed using the Adaptive Poisson Boltzmann Solver [29].

SUPPORTING FIGURES AND TABLES

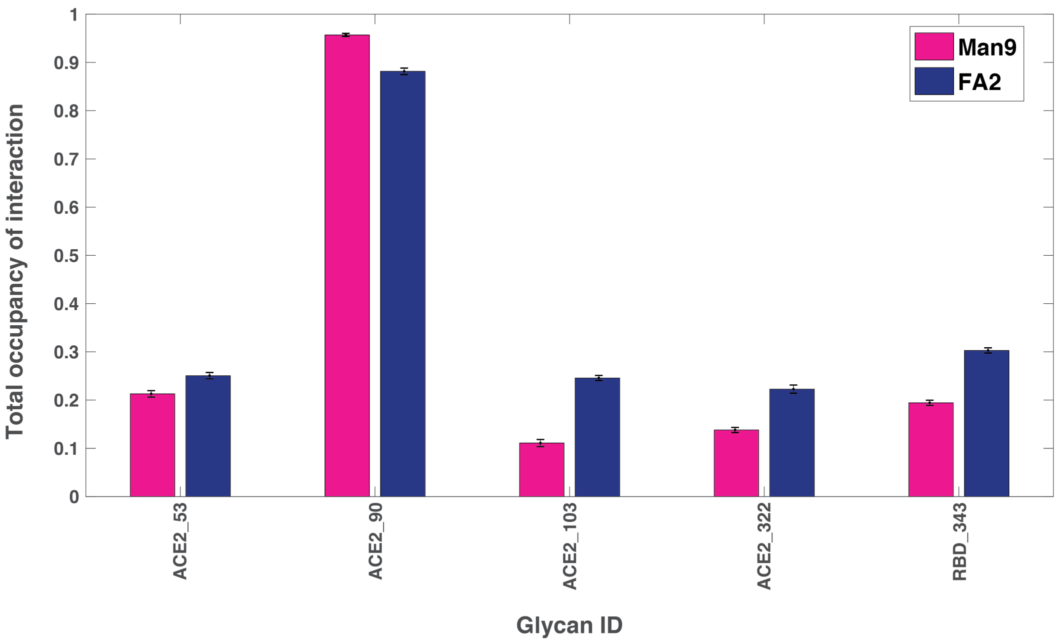

FIGURE S1. **Total probability of glycan-protein contact formation for each glycan.** Of all the glycans in ACE2, the one at position 90 contacts the RBD with distinctly higher probability (over 80%). The single RBD glycan at position 343 forms contacts with ACE2 in 20% (30%) of the sampled configurations for MAN9 (FA2). Standard deviations by bootstrapping over four sets from the total ensemble demonstrate convergence of glycan sampling.

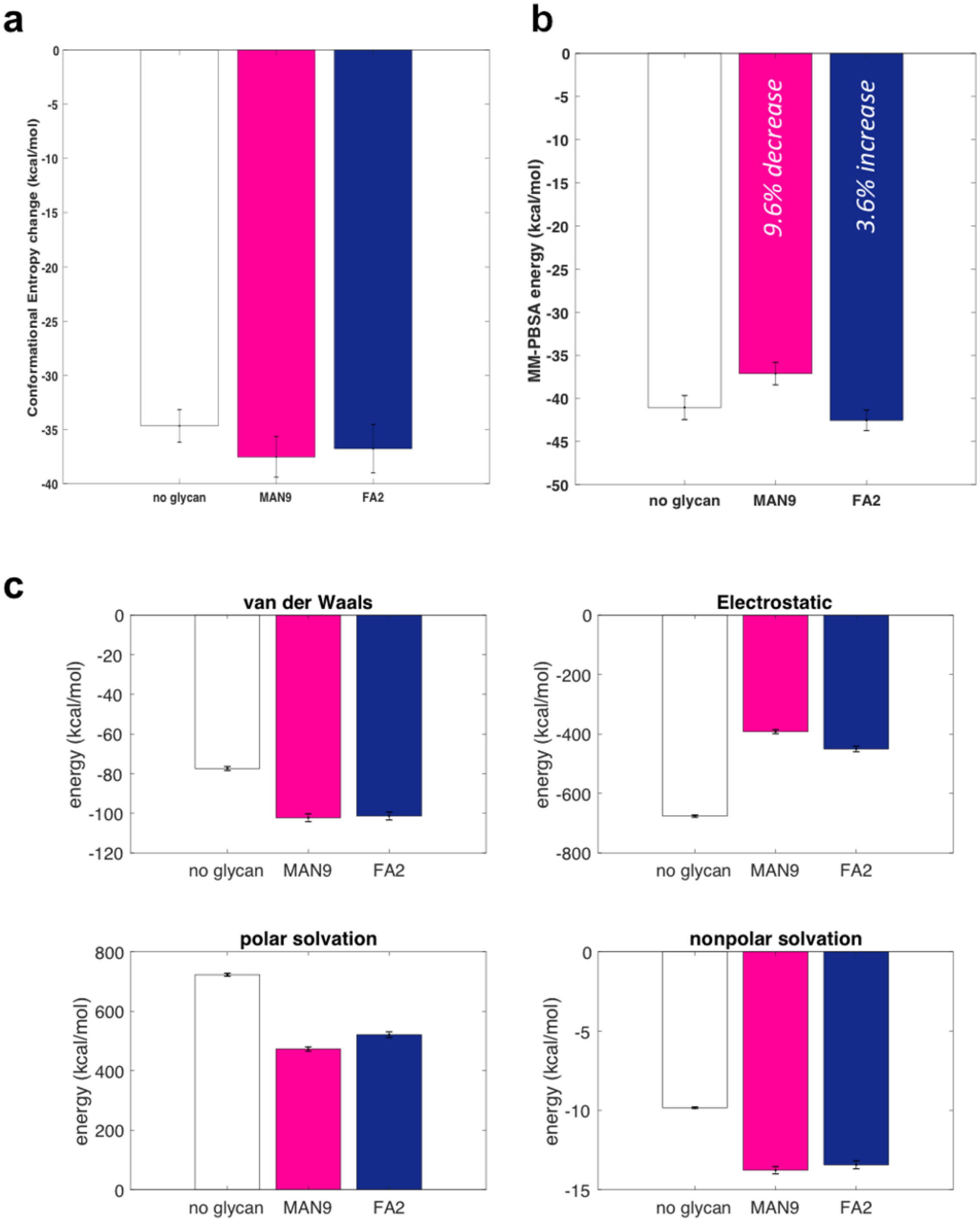

FIGURE S2. **Analysis of RBD-ACE2 binding energy. (a)** Change in conformational entropy using a quasi-harmonic approximation. **(b)** Same MM-PBSA analysis as in main Figure 5a, except that glycan Asn90 is removed from the simulations with MAN9 (magenta), or FA2 glycans (blue) in ACE2. **(c)** Decomposition of contributions to the MM-PBSA energy as shown in main Figure 5a into van der Waals, electrostatic, polar solvation, and non-polar solvation energy components.

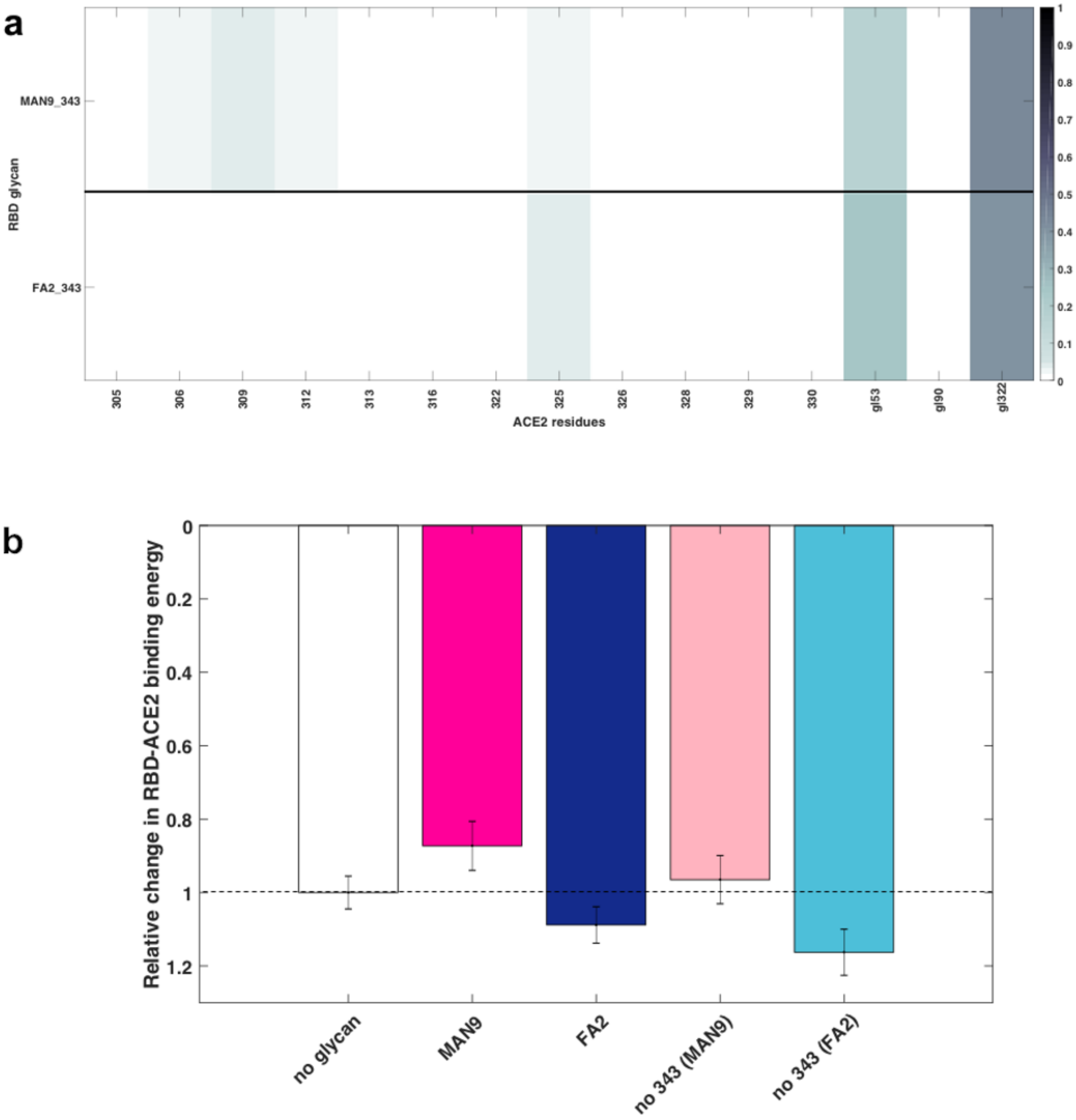

FIGURE S3. **Interactions and energetic effects associated with RBD-Asn343 glycan. (a)** Contacts of RBD-Asn343 glycan with ACE2 residues or ACE2 glycans. The RBD-Asn343 glycan forms more frequent interactions (i.e. over 20%) with glycans 53 and 322 of ACE2 (both for MAN9 and FA2). The RBD-Asn343 glycan does not form frequent contacts with ACE2 residues. **(b)** Changes in RBD-ACE2 binding energy relative to non-glycosylated RBD-ACE2. The bars of "no glycan" (white), "MAN9" (magenta), and "FA2" (blue) represent the same data as main Figure 5a, except normalized to the "no glycan" value. Recall that "MAN9" (magenta) and "FA2" (blue) correspond to the simulation sets where either MAN9, or FA2 glycans are in ACE2, respectively. In both sets, a single FA2 glycan is bound at Asn343 in RBD. To identify the energetic contribution of the RBD-Asn343 glycan, we removed this glycan and recalculated binding energies (see pink and cyan bars, which are normalized to the "no glycan" value). Specifically, the pink bar of "no 343 (MAN9)" shows RBD-ACE2 stability for the MAN9-in-ACE2 simulations, after removal of the RBD-Asn343 glycan. The cyan bar of "no 343 (FA2)" shows RBD-ACE2 stability for the FA2-in-ACE2 simulations, after removal of the RBD-Asn343 glycan.

TABLE S1: **Quantitative evaluation of contacts previously implicated by experimental structures.** RBD-ACE2 contacts found in PDB IDs: 6M0J [30], 6M17 [31], and 6VW1 [32] are listed. For each pair, the contact probably was calculated using our all-atom simulations. All persistent short-range contacts as captured by the simulations (i.e. total of 25, highlighted in green) represent a subset of the experimentally-reported interactions. Persistent short-range contacts are defined as those that form with at least 60% probability.

RBD residue	ACE2 residue	Contact Probability
417 LYS	30 ASP	0.71
446 GLY	42 GLN	0.05
449 TYR	38 ASP	0.07
449 TYR	42 GLN	0.14
453 TYR	34 HIS	0.93
455 LEU	34 HIS	0.74
456 PHE	27 THR	0.95
456 PHE	30 ASP	0.38
456 PHE	31 LYS	0.46
475 ALA	19 SER	0.17
475 ALA	24 GLN	0.76
475 ALA	27 THR	0.43
476 GLY	24 GLN	0.59
486 PHE	79 LEU	0.56
486 PHE	82 MET	0.78
486 PHE	83 TYR	0.85
487 ASN	24 GLN	0.86
487 ASN	83 TYR	0.93
489 TYR	27 THR	0.79
489 TYR	28 PHE	0.93
489 TYR	31 LYS	0.66
489 TYR	83 TYR	0.49
493 GLN	31 LYS	0.65
493 GLN	34 HIS	0.63
493 GLN	35 GLU	0.83
495 TYR	38 ASP	0.05
496 GLY	353 LYS	0.25
498 GLN	41 TYR	0.60
498 GLN	42 GLN	0.30
498 GLN	45 LEU	0.04
500 THR	41 TYR	0.85
500 THR	330 ASN	0.68
500 THR	355 ASP	0.87
500 THR	357 ARG	0.77
501 ASN	41 TYR	0.74
501 ASN	353 LYS	0.93
502 GLY	353 LYS	0.90

Continued on next page

TABLE S1 – *Continued from previous page*

RBD residue	ACE2 residue	Contact Probability
502 GLY	354 GLY	0.80
505 TYR	37 GLU	0.58
505 TYR	353 LYS	0.95
505 TYR	354 GLY	0.44
505 TYR	393 ARG	0.45

SUPPORTING REFERENCES

[1] Goh, J. B. & Ng, S. K. Impact of host cell line choice on glycan profile. *Critical Reviews in Biotechnology* **38**, 851–867 (2018). PMID: 29262720, arXiv:https://doi.org/10.1080/07388551.2017.1416577.

[2] Brooks, S. A. Appropriate glycosylation of recombinant proteins for human use: Implications of choice of expression system. *Applied Biochemistry and Biotechnology - Part B Molecular Biotechnology* **28**, 241–255 (2004).

[3] Han, D. P., Lohani, M. & Cho, M. W. Specific asparagine-linked glycosylation sites are critical for dc-sign- and l-sign-mediated severe acute respiratory syndrome coronavirus entry. *Journal of Virology* **81**, 12029–12039 (2007). arXiv:https://jvi.asm.org/content/81/21/12029.full.pdf.

[4] Feinberg, H., Mitchell, D. A., Drickamer, K. & Weis, W. I. Structural basis for selective recognition of oligosaccharides by dc-sign and dc-signr. *Science* **294**, 2163–2166 (2001). arXiv:https://science.sciencemag.org/content/294/5549/2163.full.pdf.

[5] Zhao, X. *et al.* Inhibition of endoplasmic reticulum-resident glucosidases impairs severe acute respiratory syndrome coronavirus and human coronavirus nl63 spike protein-mediated entry by altering the glycan processing of angiotensin i-converting enzyme 2. *Antimicrobial Agents and Chemotherapy* **59**, 206–216 (2015). arXiv:https://aac.asm.org/content/59/1/206.full.pdf.

[6] Shajahan, A. *et al.* Comprehensive characterization of N- and O- glycosylation of SARS-CoV-2 human receptor angiotensin converting enzyme 2. *Glycobiology* (2020). Cwaa101, arXiv:https://academic.oup.com/glycob/advance-article-pdf/doi/10.1093/glycob/cwaa101/34865695/cwaa101.pdf.

[7] Qing, E., Hantak, M., Perlman, S. & Gallagher, T. Distinct roles for sialoside and protein receptors in coronavirus infection. *mBio* **11** (2020). arXiv:https://mbio.asm.org/content/11/1/e02764-19.full.pdf.

[8] Watanabe, Y., Allen, J. D., Wrapp, D., McLellan, J. S. & Crispin, M. Site-specific glycan analysis of the SARS-CoV-2 spike. *Science* **369**, 330–333 (2020).

[9] Shajahan, A., Supekar, N. T., Gleinich, A. S. & Azadi, P. Deducing the N- and O-glycosylation profile of the spike protein of novel coronavirus SARS-CoV-2. *Glycobiology* **30**, 981–988 (2020). arXiv:https://academic.oup.com/glycob/article-pdf/30/12/981/34828309/cwaa042.pdf.

[10] Guttman, M., Weinkam, P., Sali, A. & Lee, K. All-atom ensemble modeling to analyze small-angle x-ray scattering of glycosylated proteins. *Structure* **21**, 321 – 331 (2013).

[11] Eswar, N. *et al.* Comparative protein structure modeling using modeller. *Current Protocols in Bioinformatics* **15**, 5.6.1–5.6.30 (2006). arXiv:https://currentprotocols.onlinelibrary.wiley.com/doi/pdf/10.1002/0471250953.bi0506s15.

[12] Huang, J. *et al.* CHARMM36m: An improved force field for folded and intrinsically disordered proteins. *Nature Methods* **14**, 71–73 (2016).

[13] Berndsen, Z. T. *et al.* Visualization of the hiv-1 env glycan shield across scales. *Proceedings of the National Academy of Sciences* **117**, 28014–28025 (2020). arXiv:https://www.pnas.org/content/117/45/28014.full.pdf.

[14] Chakraborty, S. *et al.* Quantification of the resilience and vulnerability of hiv-1 native glycan shield at atomistic detail. *iScience* **23**, 101836 (2020).

[15] Case, D. A. *et al.* Amber 16 (2016). University of California, San Francisco.

[16] Case, D. A. *et al.* The amber biomolecular simulation programs. *Journal of Computational Chemistry* **26**, 1668–1688 (2005). arXiv:https://onlinelibrary.wiley.com/doi/pdf/10.1002/jcc.20290.

[17] Srinivasan, J., Cheatham, T. E., Cieplak, P., Kollman, P. A. & Case, D. A. Continuum solvent studies of the stability of DNA, RNA, and phosphoramidate-DNA helices. *Journal of the American Chemical Society* **120**, 9401–9409 (1998).

[18] Kollman, P. A. *et al.* Calculating structures and free energies of complex molecules: Combining molecular mechanics and continuum models. *Accounts of Chemical Research* **33**, 889–897 (2000).

[19] Miller, B. R. *et al.* MMPBSA.py: An efficient program for end-state free energy calculations. *Journal of Chemical Theory and Computation* **8**, 3314–3321 (2012).

[20] Honig, B. & Nicholls, A. Classical electrostatics in biology and chemistry. *Science* **268**, 1144–1149 (1995). arXiv:https://science.sciencemag.org/content/268/5214/1144.full.pdf.

[21] Wong, S., Amaro, R. E. & Andrew McCammon, J. MM-PBSA captures key role of intercalating water molecules at a protein-Protein interface. *Journal of Chemical Theory and Computation* **5**, 422–429 (2009).

[22] Genheden, S. *et al.* Accurate predictions of nonpolar solvation free energies require explicit consideration of binding-site hydration. *Journal of the American Chemical Society* **133**, 13081–13092 (2011).

[23] Mishra, S. K. & Koča, J. Assessing the Performance of MM/PBSA, MM/GBSA, and QM-MM/GBSA Approaches on Protein/Carbohydrate Complexes: Effect of Implicit Solvent Models, QM Methods, and Entropic Contributions. *Journal of Physical Chemistry B* **122**, 8113–8121 (2018).

[24] Chong, S. H. & Ham, S. Dissecting Protein Configurational Entropy into Conformational and Vibrational Contributions. *Journal of Physical Chemistry B* **119**, 12623–12631 (2015).

[25] Wang, J., Morin, P., Wang, W. & Kollman, P. A. Use of MM-PBSA in reproducing the binding free energies to HIV-1 RT of TIBO derivatives and predicting the binding mode to HIV-1 RT of efavirenz by docking and MM-PBSA. *Journal of the American Chemical Society* **123**, 5221–5230 (2001).

[26] Xu, D. *et al.* Distinct glycan topology for avian and human sialopentasaccharide receptor analogues upon binding different hemagglutinins: A molecular dynamics perspective. *Journal of Molecular Biology* **387**, 465 – 491 (2009).

[27] Ieong, P., Amaro, R. & Li, W. Molecular dynamics analysis of antibody recognition and escape by human h1n1 influenza hemagglutinin. *Biophysical Journal* **108**, 2704 – 2712 (2015).

[28] Van Der Spoel, D. *et al.* Gromacs: Fast, flexible, and free. *Journal of Computational Chemistry* **26**, 1701–1718 (2005). arXiv:https://onlinelibrary.wiley.com/doi/pdf/10.1002/jcc.20291.

[29] Baker, N. A., Sept, D., Joseph, S., Holst, M. J. & McCammon, J. A. Electrostatics of nanosystems: Application to microtubules and the ribosome. *Proceedings of the National Academy of Sciences* **98**, 10037–10041 (2001). arXiv:https://www.pnas.org/content/98/18/10037.full.pdf.

[30] Lan, J. *et al.* Structure of the SARS-CoV-2 spike receptor-binding domain bound to the ACE2 receptor. *Nature* **581**, 215–220 (2020).

[31] Yan, R. *et al.* Structural basis for the recognition of SARS-CoV-2 by full-length human ACE2. *Science* **367**, 1444–1448 (2020).

[32] Shang, J. *et al.* Structural basis of receptor recognition by SARS-CoV-2. *Nature* **581**, 221–224 (2020).
